# The three-dimensional plasma structures and flows of the Earth’s upper atmosphere due to the Moon’s gravitational force

**DOI:** 10.1038/s41598-022-25449-y

**Published:** 2022-12-05

**Authors:** Jann-Yenq Liu, Tsung-Yu Wu, Chi‐Yen Lin, Loren C. Chang

**Affiliations:** 1 Center for Astronautical Physics and Engineering, National Central University, Taoyuan, Taiwan; 2 Department of Space Science and Engineering, National Central University, Taoyuan, Taiwan; 3 Center for Space and Remote Sensing Research, National Central University, Taoyuan, Taiwan

**Keywords:** Space physics, Atmospheric science

## Abstract

The semidiurnal (12.42 h) and semimonthly (14.76 days) lunar tides have been well-known by fishermen for several centuries. The gravitational force of the relative positions between the Sun, the Moon, and the Earth results in two symmetrical tidal bulges (double bulges) appearing at equatorial latitudes directly under and opposite the Moon. We utilize ionospheric GNSS (Global Navigation Satellite System) radio occultation soundings to show the global three-dimensional structures and dynamics of the double bulges of ionospheric lunar tides for the first time. The double-bulge amplitude of ionospheric F2-peak height hmF2, lagging the sublunar or antipodal point by about 2–3 h, is about 3–5 km at the equator and 1.5–2.0 km at ± 35° magnetic latitude. The electron density further depicts global three-dimensional plasma flows in the ionosphere.

## Introduction

For thousands of years, human beings, especially those of maritime trades such as sailors and fishermen, have known that the oceanic surface height (i.e., tidal bulge, high tide) yields prominent variations with a period of about 12.42 h, termed the semidiurnal lunar tide^1^ due to the Moon’s gravitational force and the Earth’s rotation. The lowest oceanic surface height between the two tidal bulges is termed low tide. The span of time that it takes for the Moon’s disk to change from all dark to all light to all dark again is called the lunar month or phase, which is about 29.53 days. This oceanic surface height further varies according to the lunar phase of new moon, first quarter, full moon, and third quarter, which reflects the relative positions between the Sun, the Moon, and the Earth. When the three objects line up during either new moon or full moon, the lunar tide attains maximum amplitude. The time interval between the next adjacent maximum tide is one half of the moon’s revolution period of 14.76 days, which has been also observed for several centuries and is known as the semimonthly lunar tide. Based on the Equilibrium Tide developed from Newton's theory of gravitation, two symmetrical tidal bulges of the sea level should appear with amplitudes of about 0.5 m at equatorial latitudes directly under and directly opposite the moon^1^. However, for the semidiurnal tide, the times of tidal high water can vary due to global continental distribution, as the tides spread from the oceans onto the surrounding continental shelves, bearing no simple relationship to the ideal double bulge^1,2^.

By contrast, since the upper atmosphere is essentially a continuous fluid without the continental pattern effect superimposed, the free surface of the Earth’s ionosphere should respond by adapting to the shape of the Equilibrium Tide^1^. Unlike the ocean water with a constant density of about 1 g/cm^3^, the atmospheric density varies based on hydrostatic equilibrium and the ideal gas law, decreasing exponentially with altitude, which results in the atmospheric density at about 300 km altitude being about one thousand billion times smaller than that on the ground^3^. Although semidiurnal lunar tide signatures of electron density, wind, temperature, etc. in the upper atmosphere and ionosphere at certain regions, zones, and times have been reported^4–9^, due to the lack of global and uniform three-dimensional (3D) observations, the response of the ionospheric F2-peak height (hmF2) to the double bulge of upper atmospheric lunar tides owing to the Moon’s gravitational force has not yet been globally observed.

## Radio occultation data and 3D observation

**Figure 1:**
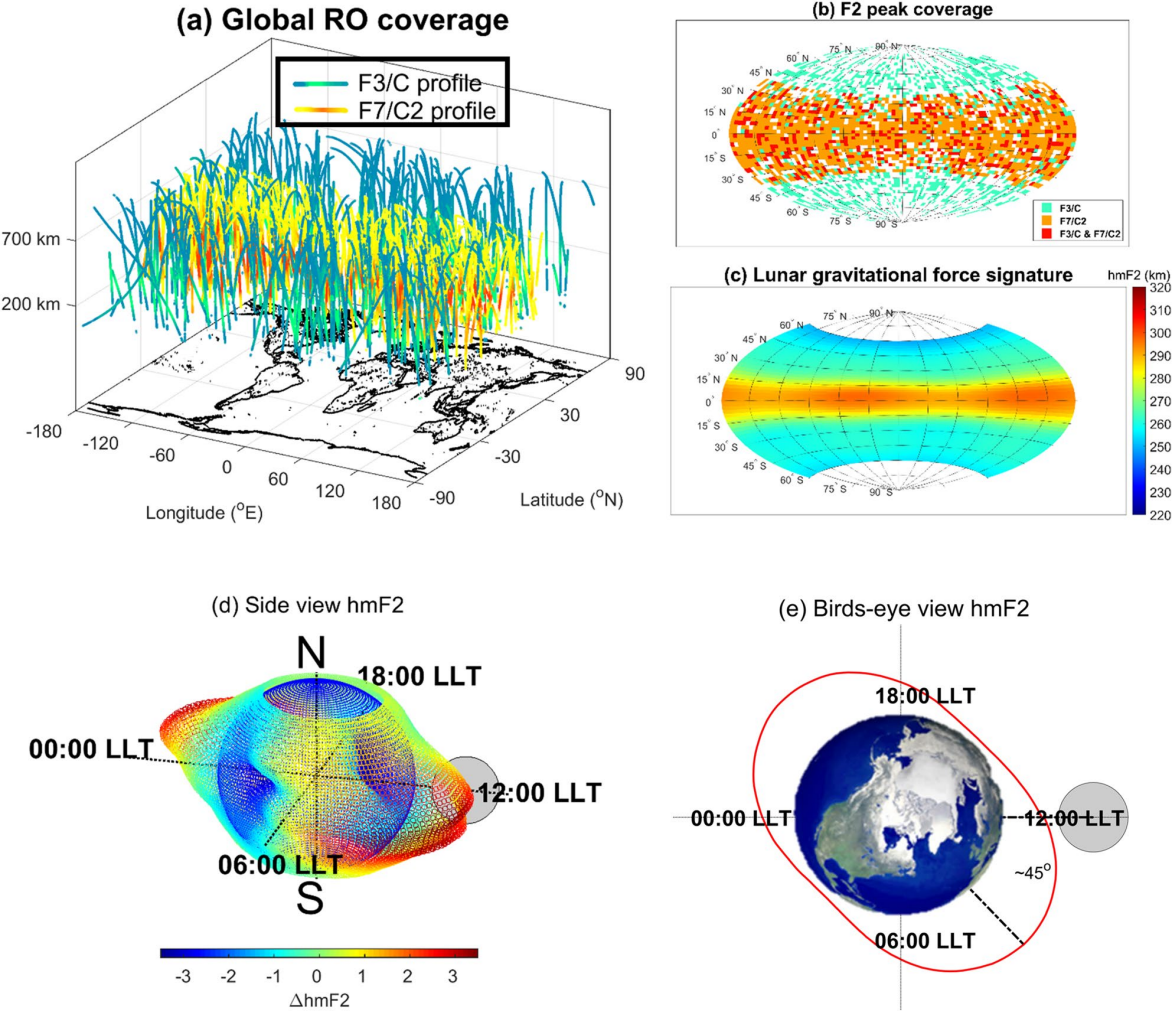
F3/C and F7/C2 ionospheric RO profiles. (**a**) Locations of daily F3/C and F7/C2 RO observations. (**b**) Locations of daily F3/C and F7/C2 F2-peak observations. (**c**) The global ionospheric F2-peak height (hmF2) on the magnetic latitude-lunar local time plane, as well as its (**d**) side-view (spherical view), and (**e**) birds-eye (equatorial plane) view over the entire 15-year composite. The red ellipse denotes the meridional averaged hmF2. The gray circle denotes the Moon position. The Global View of the Arctic Ocean is published by NASA/JPL/ASF (https://www.jpl.nasa.gov/images/pia02970-global-view-of-the-arctic-ocean).

The FORMOSAT-3/COSMIC (Constellation Observing System for Meteorology, Ionosphere & Climate, F3/C) constellation, consisting of six micro-satellites, was the world’s first satellite constellation dedicated to Global Positioning System radio occultation (GPS-RO), demonstrating the value of GPS-RO data in climate, atmosphere weather, and space weather studies^10–13^ (Supplementary Fig. S1). The F3/C GPS-RO soundings provided over 6.9 million atmospheric temperature and water vapor pressure profiles from the ground to about 40 km altitude, and 4.6 million ionospheric electron density profiles from 100 to 800 km altitude during 2006–2020 (Supplementary Fig. S2). Following the success of F3/C, FORMOSAT-7/COSMIC-2 (F7/C2), consisting of an additional six small satellites, was successfully launched on 25 June 2019 and has been conducting GNSS (Global Navigation Satellite System) RO observations (Supplementary Fig. S3), which have been producing daily soundings of more than 4,000 atmospheric and ionospheric profiles over the tropics (Fig. 1a,b, Supplementary Fig. S4). In total, 5,776,061 RO electron density profiles by F3/C and F7/C2, 4,512,117 F3/C profiles during 21 April 2006–25 April 2020 and 1,263,944 F7/C2 profiles during 16 July 2019–2 November 2020, are utilized to globally examine the 3D ionospheric (or upper atmospheric) electron density structures and variations in response to the lunar phase during the 15-year period of 2006–2020.

## Results

The 5,776,061 electron density profiles are examined to find the response of hmF2 and NmF2 to lunar gravitational force signatures during 2006–2020. Based on the Lunar Calendar (https://www.timeanddate.com/), the associated lunar local time (LLT) of each hmF2 or NmF2 is obtained. To extract lunar phase (i.e., gravitational force) signatures, we construct Fig. 1c–e by binning hmF2 into a magnetic latitude-LLT grid and then averaging over the entire 15 years to form a composite global surface (Fig. 1c). The spherical (Fig. 1d) and equatorial plane (Fig. 1e) views in the equatorial and low latitude ionosphere for the first time show that a clear double bulge with about 3 km amplitude of hmF2 at about 270-km altitude lags the point directly under and directly opposite the Moon by about 45 degrees in LLT, which is equivalent to ~ 3 h in the semidiurnal lunar tide or ~ 3.7 days in the semimonthly lunar tide.

**Figure 2:**
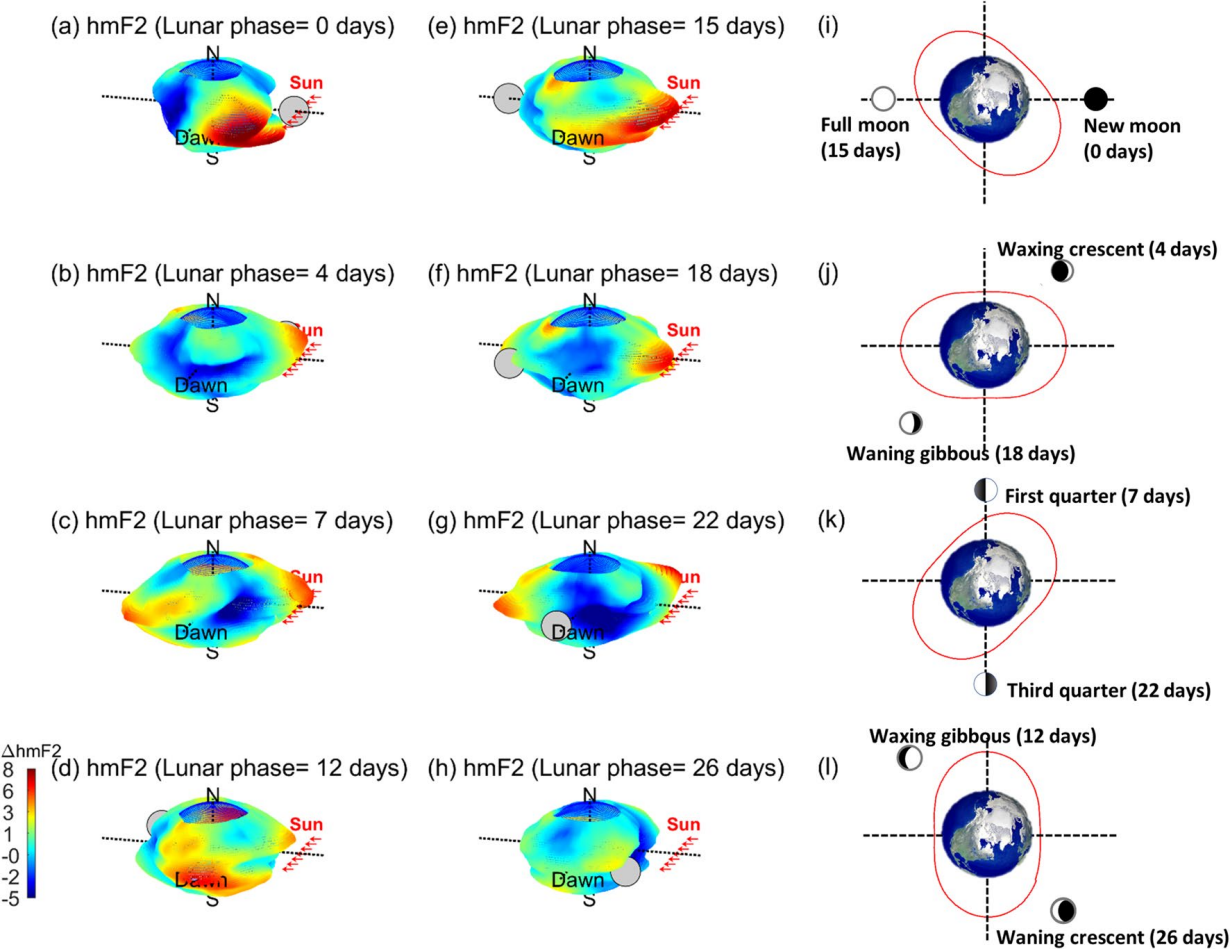
Global composite hmF2 during lunar phases of the (**a**) new moon (0 days), (**b**) waxing crescent (4 days) (**c**) first quarter (7 days), (**d**) waxing gibbous (12 days), (**e**) full moon (15 days), (**f**) waning gibbous (18 days), (**g**) third quarter (22 days), and (**h**) waning crescent (26 days). (**i–l**) Sketches of hmF2 on the equatorial plane on the 0/15, 4/18, 7/22, and 12/26 days. The red ellipse denotes the meridional averaged hmF2. The gray circle denotes the Moon position.

Astronomers have further sub-divided the lunar month/phase of 29.53 days into eight primary Moon phases: new moon (0 days), first quarter (7 days), full moon (15 days), third quarter (22 days), waxing crescent (4 days), waxing gibbous (12 days), waning gibbous (18 days), and waning crescent (26 days). To study the response of hmF2 to the lunar gravitational force during each Moon phase, we construct Fig. 2a–h by binning hmF2 into a magnetic latitude-LLT grid based on the eight Moon phases and then averaged over the entire 15 years. It can be seen that the double bulge patterns are similar on new moon/full moon (Fig. 2a,e and Supplementary Fig. S5a,e), first quarter/third quarter (Fig. 2c,g, Supplementary Fig. S5c,g), waxing crescent/waning gibbous (Fig. 2b,f, Supplementary Fig. S5b,f), and waxing gibbous/waning crescent (Fig. 2d,h, Supplementary Fig. S5d,h), respectively. This again confirms that the lunar gravitational force leads the hmF2 double bulges by about 3 h in the semidiurnal lunar tide or 3.7 days in semimonthly lunar tide (Figs. 1d,e, 2a–h).

**Figure 3:**
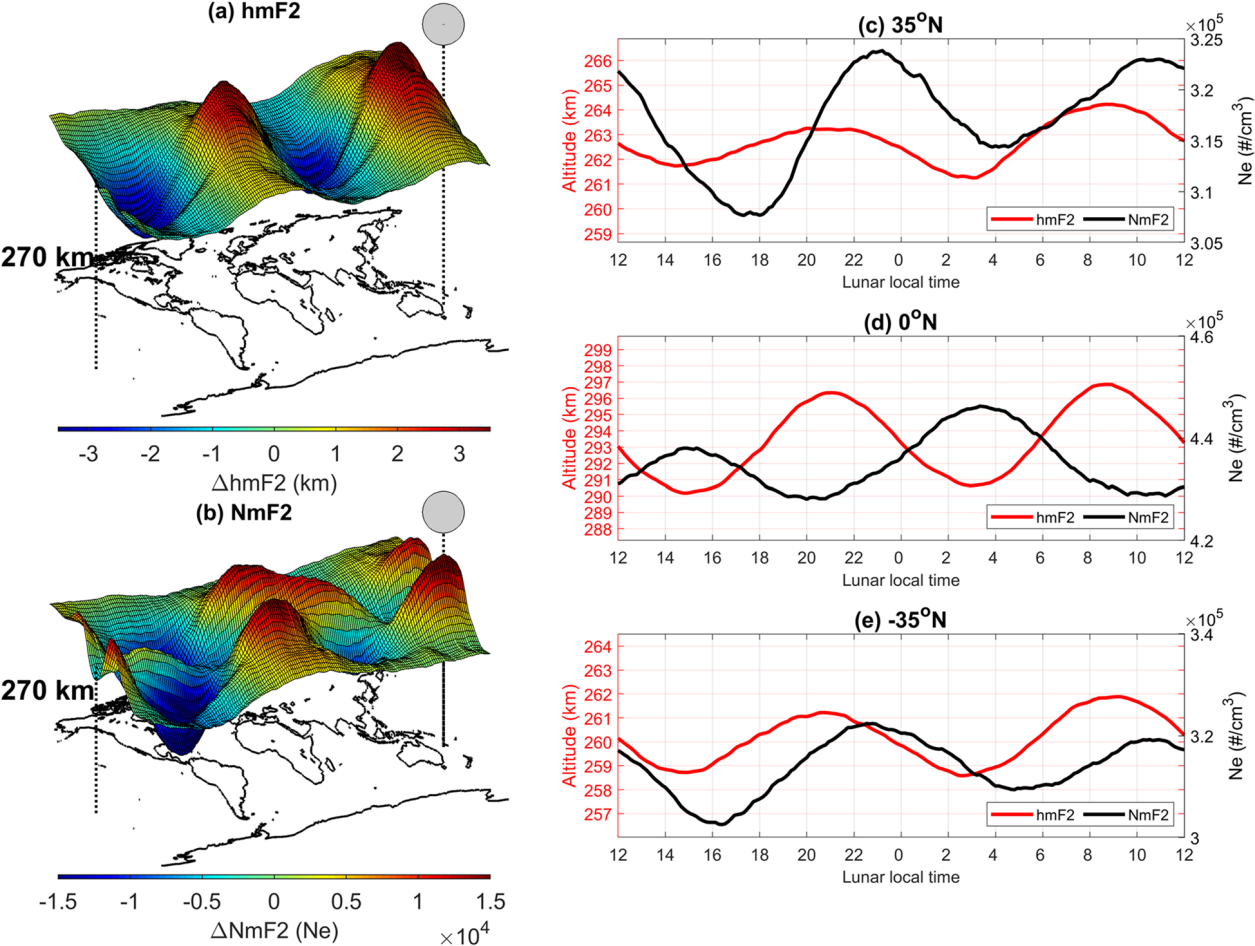
Global 3D F3/C and F7/C2 F2-peak. (**a**) pseudo hmF2 and (**b**) pseudo NmF2 on the map of ± 60° N magnetic latitude and ± 180° E longitude with the sublunar point (i.e., 12:00 lunar local time) at 0° N magnetic latitude and 180° E longitude. hmF2 (red curves) and NmF2 (black curves) at (**c**) 35° N, (**d**) 0° N, and (**e**) − 35° N magnetic latitude.

In addition to hmF2, we further bin NmF2, which is the ionospheric F2-peak electron density, into a magnetic latitude-LLT grid and average the values over the entire 15 years. We then examine hmF2 and NmF2 within ± 60° magnetic latitude, as well as at the magnetic equator and ± 35° magnetic latitude at various LLTs. The sublunar point corresponds to 12:00 LLT. Here, hmF2 simultaneously reaches a peak within ± 60° magnetic latitudes at 09:00 LLT (Fig. 3a and red curves of Fig. 3c–e), while NmF2 shows a peak around the magnetic equator at 15:00 LLT and around ± 35° magnetic latitude at 11:00 LLT (Fig. 3b and black curves of Fig. 3c–e). Similar to Figs. 1e and 2a–h, hmF2 constantly lags the sublunar point at various magnetic latitudes by about 3–4 h (Fig. 3a and red curves of Fig. 3c–e). NmF2 and hmF2 are approximately in antiphase (about 6 h) at the magnetic equator, while the NmF2 leads the hmF2 by 2 h at ± 35° N magnetic latitude. Therefore, NmF2 at the magnetic equator leads that at ± 35° N magnetic latitude by about 4 h. The discrepancy in the time difference between NmF2 and hmF2 at the magnetic equator and ± 35° N magnetic latitude suggests that the 3D plasma transport is important. Nevertheless, Fig. 3 once again shows that the ionospheric double bulge of hmF2 lags the sublunar or antipodal point by about 3 h in the semidiurnal lunar period or 3.7 days in the semimonthly lunar period. The double bulge signatures are further investigated with and without magnetic storms (Dst > − 50 nT) as well as under high (F10.7 ≥ 100 sfu) and low solar (F10.7 < 100 sfu) activities. It might be a magnetic storm period usually is shorter than the semimonthly period, and therefore no obvious magnetic storm effects on NmF2 and hmF2 can be observed (Supplementary Fig. S6). On the other hand, hmF2 ascends to higher altitudes and NmF2 reach greater values, both slightly expend toward higher latitudes during the high solar activity (Supplementary Fig. S6). Nevertheless, prominent double bulge features can be observed under virous magnetic conductions and solar activities.

**Figure 4:**
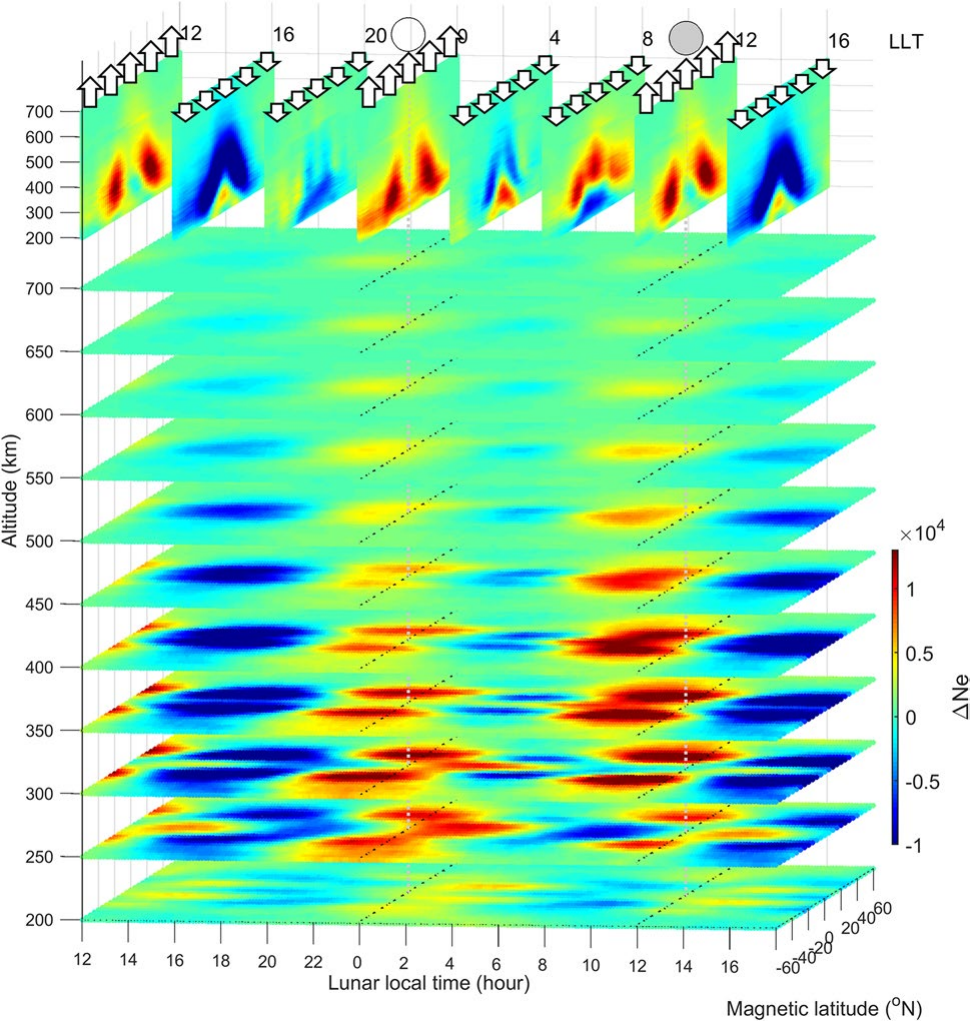
Composite 3D electron density structure observed by the F3/C and F7/C2 at various lunar local time during 2006–2020. Longitudinal slices every 3 LLT hours are shown at the top. Open arrows stand for lunar gravitational forces. The gray circle denotes the Moon position, while the white circle is the antipodal point.

Since the F3/C and F7/C2 3D electron density observations are available, we then examine the response of the upper atmospheric circulation to the lunar gravitational force by binning the electron density into a magnetic latitude-altitude-LLT grid and then averaging over the entire 15 years (Fig. 4, Supplementary Fig. S7). For horizontal slices below 300 km altitude, the electron density increase or decrease resembles a flat “Y” character shape, with the foot of the “Y” pointing forward in LTT, over the high (i.e., bulge) or low (i.e., between the two bulges) tide sector. For those above 350 km altitude, two dense and rarefied electron density bands centered at ± 25° N magnetic latitude but straddling the equator appear at the high tide and low tide sectors, respectively. Magnetic latitude-altitude slices reveal that the dense or rarefied electron density shows a shape similar to the Greek capital letter Lamda “Λ” on either high or low tide sectors, with the letter feet at 25° and − 25° N magnetic latitude and the letter apex at about above 500 km altitude around the equator.

## Discussion

Previous studies show that vertical forces due to the lunar gravitational force can simply produce small changes in the weight of a fluid parcel, but it is the small horizontal forces from the continuity that produce the tidal accelerations necessary to drive the tidal movements in the ocean^1^. Thus, owing to the conservation of mass and the divergence theory, the maximum in the vertical upward velocity right under the sublunar or antipodal points (i.e., 12:00 LLT or 00:00 LLT) are in phase with that in the horizontal velocity convergence at the midway between the two points (i.e., 06:00 LLT or 18:00 LLT). Based on simple harmonic motion (SHM), the maximum vertical displacement should lag the vertical velocity by about 90°, or a quarter cycle^14^. The quarter cycle of the 12.42-h semidiurnal or 14.76-day semimonthly lunar periods agrees well with the hmF2 double bulges, lagging the sublunar or antipodal points by about 3 h for the semidiurnal lunar period or 3.7 days for the semimonthly lunar period (Figs. 1, 2, 3, 4).

**Figure 5:**
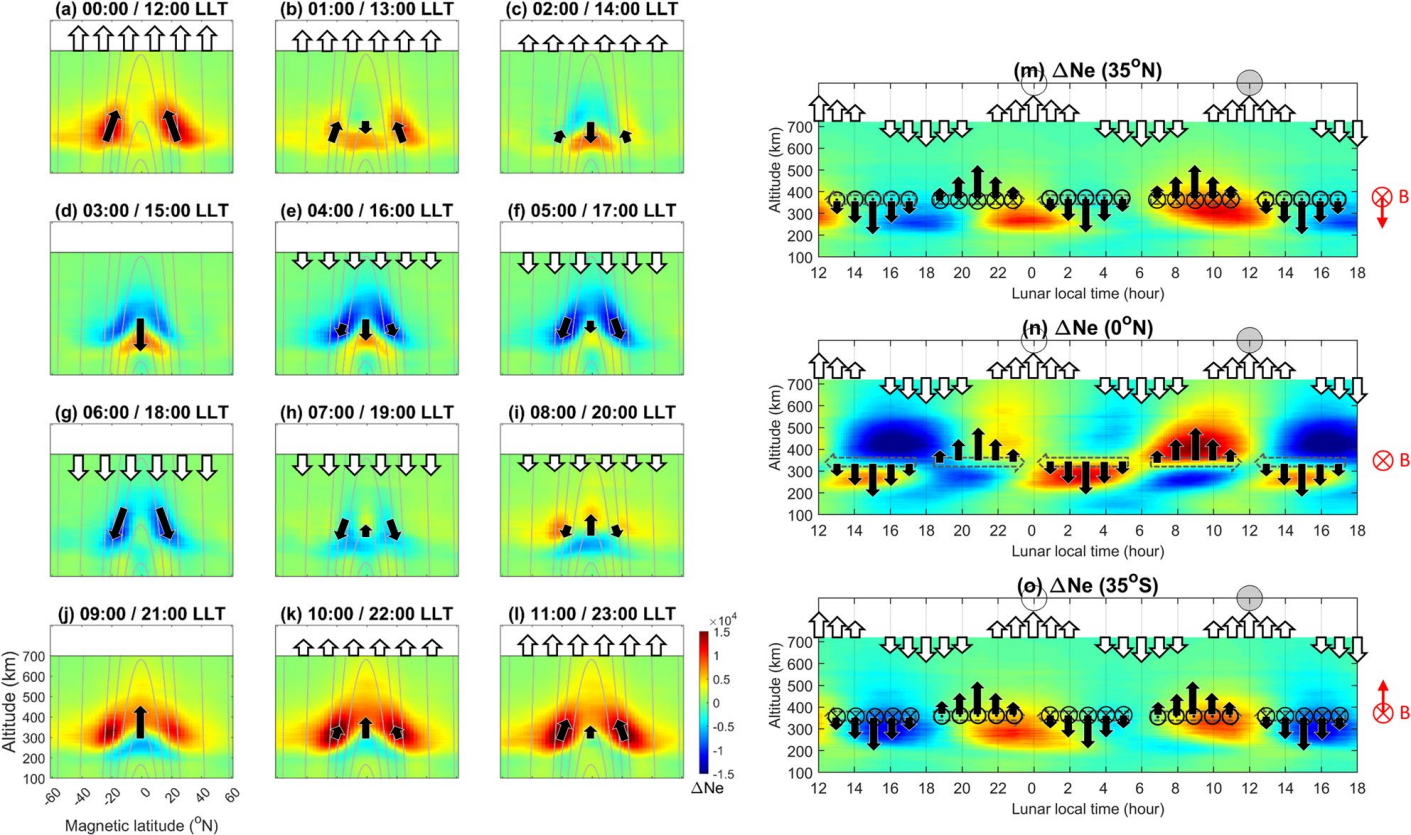
(**a**–**l**) Altitude-magnetic latitude slices F3/C and F7/C2 electron density, where the gray curves denote the magnetic field, and the black arrows stand for bulk plasma motions. Altitude-lunar local time slices at the (**m**–**o**). magnetic equator and ± 35° N magnetic latitudes. The Earth’s magnetic field is in the inward direction at the magnetic equator and in the up-inward/down-inward direction at ± 35° N magnetic latitudes. Dashed arrows stand for responses of eastward and westward horizontal neutral winds to the lunar gravitational force. Therefore, black arrows denote plasma drifts driven by the neutral wind.

Similarly, based on the continuity equation and SHM^15^, the hmF2 and NmF2 maximum respectively lag and lead the sublunar or antipodal points by 90° at the magnetic equator, which corresponds well with hmF2 and NmF2 being in antiphase at the magnetic equator (Fig. 3d). Figure 3c–e reveal that there is a time delay of 4 h in NmF2 between the magnetic equator and ± 35° N magnetic latitude. Since Figs. 1, 2, 3 and 4 show two semidiurnal (or semimonthly) lunar periods being nearly identical, we then superimpose the two periods and examine the electron density variation to have a better understanding of the response of upper atmospheric circulation to the lunar gravitational force (or LLT) in detail (Fig. 5, Supplementary Fig. S8; Supplementary Video 1). For the meridional plane (i.e., altitude-magnetic latitude slice) at about 10:00–14:00 LLT, the lunar gravitational force triggers upward and equatorward air/plasma motions along the magnetic field line due to atmospheric neutral drag (Fig. 5k,l;a–c), where the upward and equatorial motions reach the maximums at 12:00 LLT (i.e., the sublunar point). At about 18:00 LLT, downward and poleward air/plasma motions are resolved, extending between 16:00 and 20:00 LLT (i.e., between the sublunar and antipodal points) (Fig. 5e–i). The upward motion results in the plasma increasing and decreasing above and below the F2-peak, respectively (Supplementary Fig. S9a,b). Vice versa, the downward motion causes the plasma to decrease (increase) above (below) the F2-peak (Supplementary Fig. S9c,d). Figure 5m–o show altitude-LLT slices at the magnetic equator and ± 35° magnetic latitudes. The lunar gravitational force can be seen to induce eastward and westward neutral wind motions ($$ \overset{\lower0.5em\hbox{$\smash{\scriptscriptstyle\rightharpoonup}$}}{U}  $$) at 07:00–11:00 (19:00–23:00 LLT) and 13:00–17:00 (01:00–05:00 LLT), respectively. Owing to the $$ {\text{q}}\overset{\lower0.5em\hbox{$\smash{\scriptscriptstyle\rightharpoonup}$}}{U}  \times \overset{\lower0.5em\hbox{$\smash{\scriptscriptstyle\rightharpoonup}$}}{B}  $$ Lorentz force, the plasma exhibits downward drift at 13:00–17:00 (01:00–05:00 LLT) with maxima at 15:00 (03:00 LLT) (Fig. 5b–f,m–o), and upward drift at 07:00–11:00 (19:00–23:00 LLT) with maxima at 09:00 (21:00 LLT) (Fig. 5h–l,m–o). Note that there are poleward and equatorward components respectively associated with the downward and upward drifts at ± 35° N magnetic latitudes (Fig. 5m,o).

The maximum upward bulk plasma motion occurs at 00:00 (12:00 LLT) (Fig. 5a) and the maximum upward plasma drift occurs at 21:00 (09:00 LLT) (Fig. 5m–o). This is then followed by downward diffusion along magnetic field lines from the equator to off-equator regions, with the electron density showing maximum at ± 35° N magnetic latitude at 22:30 (10:30 LLT) (black curves of Figs. 3c,e, 4). By contrast, the maximum downward plasma motions occur at 06:00 (18:00 LLT) (Fig. 5g) and the maximum downward plasma drift occurs at 03:00 (15:00 LLT) (Fig. 5m–o). The plasma yields minimum at ± 35° N magnetic latitude at 04:30 (16:30 LLT) (black curves of Figs. 3c,e, and 4). The downward plasma drifts can also significantly result in the dense band of electron density of the letter foot of the “Y”-shaped horizontal structure along the magnetic equator during 13:00–16:00 LLT (Figs. 3b–e, 4), while the upward plasma drifts cause the rarefaction band during 07:00–11:00 LLT (Figs. 3b–e, 4).

Owing to the continental distribution^1,2^, a global double bulge structure in the ocean surface has not yet been observed. By contrast, the continuous upper atmosphere provides the best opportunity to observe two lunar tidal bulges on a global scale. The 3 km tidal bulges of hmF2 are about 2000 times greater than that of the sea level of 0.5 m at equatorial latitude^1^, which is indicative of the difference in density between air and water. On the other hand, the high tide of the sea surface tends to lead the sublunar point by about 2 hours^1^, while hmF2 constantly lags the sublunar point by about 3 h, which shows the friction of the ocean bottom playing an important role, and that played by the lower boundary of the upper atmosphere being much less significant. Note also that the maximum depth of the ocean is about 11 km^1,2^, while the ionosphere in this study ranges from 100 to 700 km altitude. Time variations of the electron density show that the plasma transport is driven by the force of neutral drag^3^ and the $$ {\text{q}}\overset{\lower0.5em\hbox{$\smash{\scriptscriptstyle\rightharpoonup}$}}{U}  \times \overset{\lower0.5em\hbox{$\smash{\scriptscriptstyle\rightharpoonup}$}}{B}  $$ Lorentz force. In conclusion, more than 4.6 million electron density profiles observed by the FORMOSAT‐3/COSMIC and FORMOSAT-7/COSMIC-2 have been used to examine the 3D electron density structures and variations (i.e., plasma flows and drifts), uncovering the double bulges of the upper atmosphere due to the lunar gravitational force for the first time.

## Methods

### Radio occultation

Global Positioning System Radio Occultation (GPSRO), nowadays also known as Global Navigation Satellite System Radio Occultation (GNSSRO), is a type of radio occultation that relies on radio transmissions from the U.S. GPS (Global Positioning System), or more generally from GNSS (global navigation satellite system), satellites^16,17^. This is a relatively new technique first applied in 1995 during the Global Positioning System/Meteorology (GPS/MET) mission^17,18^ for performing Earth atmospheric measurements. The technique involves a low-Earth orbit (LEO) satellite (such as MicroLab-1^17,18^, FORMOSAT-3/COSMIC, FORMOSAT-7/COSMIC-2^19,20^) receiving signals from a GPS/GNSS satellite. The signal has to pass through the ionosphere and is time-delayed along the way (Supplementary Fig. S2). The magnitude of the time delay depends on the integrated electron density or total electron content in the ionosphere^15^. GNSS radio occultation amounts to an almost instantaneous measurement of the atmospheric state. The relative position between the GPS/GNSS satellite and the LEO satellite changes over time, allowing for a vertical scanning of successive layers of the ionosphere. If we assume that the electron density is constant within each layer and that the ionosphere has a spherical symmetry (principle of onion peeling) (Supplementary Fig. S10), then through the Abel transform^21^ we can obtain a vertical profile of the electron density from 100 km to satellite altitude. Usually, the F2-peak electron density (NmF2) and height (hmF2) of each RO profile have been denoted (Fig. 1, Supplementary Fig. S4). The electron density profiles retrieved from RO measurements have been validated previously^22–27^. F3/C orbits at 800 km altitude observed 4,512,117 electron density profiles during 21 April 2006–25 April 2020, while F7/C2 orbits at 550 km altitude sounded 1,263,944 profiles during 16 July 2019–2 November 2020. In total, there are 5,776,061 electron density profiles from 100 to 800 km altitude during 2006–2020. Meanwhile, Liu et al.^13^ show that the assumption of spherical symmetry introduces artificial plasma cave and plasma tunnel structures as well as the electron density enhancement at the magnetic equator at and below 250 km altitude during daytime. Therefore, we mainly focus on NmF2, hmF2, and electron density profiles within 200–500 km altitude.

### Lunar local time and lunar phase

Lunar local time (LLT) is a calculation of the angle between the Moon and a specific meridional plane of the Earth. One lunar day represents a complete cycle that the Moon passes through 360° and returns to an Earth’s specific meridional plane. A lunar day is further divided into 24 lunar hours (15° = 1 h). 00:00 LLT stands for the antipodal of the Moon on the Earth, and 12:00 LLT means the Moon is directly overhead. Lunar phases are the relative positions between the Sun, the Moon, and the Earth. Based on the Lunar Calendar (https://www.timeanddate.com/), the associated LLT and lunar phase of each observable can be obtained. Meanwhile, astronomers have subdivided the lunar month/phase of 29.53 days into eight primary Moon phases: new moon (0 days), first quarter (7 days), full moon (15 days), third quarter (22 days), waxing crescent (4 days), waxing gibbous (12 days), waning gibbous (18 days), and waning crescent (26 days).

### Lunar phase signatures

Based on the spirit of coherent integration^9,28–30^, any periodicities other than the lunar day period are averaged out, and the signal-to-noise ratio of signatures due to lunar gravitational force is enhanced. There are 5,776,061 electron density profiles and about 5,000 lunar days during 2006–2020, which provides an excellent opportunity to conduct coherent integration studies. To study the lunar phase signatures, the magnetic latitude-LLT map with 1,176 lattices (= 49*24), 49 grids from − 60° N to 60° N magnetic latitude (2.5° per grid) and 24 grids for a lunar day, is ideally employed. The magnetic latitudes are calculated by APEX coordinate conversion^31^ published by NOAA. Here, hmF2 and NmF2 at the same magnetic latitude-LLT grid are binned and averaged over to find the lunar phase (or gravitational) signature. Taking the lunar location as the reference, the magnetic latitude-LLT maps of hmF2 and NmF2 are constructed (Figs. 1, 3). Figure 2a–h are constructed by a similar process but further subdivided into lunar phases 0–29 days, and therefore, the number of data points of each figure is about 1/30 of Fig. 1. Thus, for overall, the number of measurements per bin of Fig. 1c–e is 4,911 (= 5,776,061/1,176) profiles. For each lunar day, Fig. 2 is constructed by a similar process but subdivided into lunar phases 0–29 days. Consequently, the number of data points per bin of each Fig. 2 is about 160 (≈ 5,776,061/1,176/30). Similarly, the electron density at each altitude is dependently binned into the associated magnetic latitude-altitude-LLT lattice, then averaged over the entire 15-year period of 2006–2020. Thus, the 3D electron density at 200–700 km altitude with an interval of 2 km is obtained (Figs. 4, 5).

## Supplementary Information

Supplementary Information 1.

Supplementary Information 2.

## Data Availability

The electron density of FORMOSAT-3/COSMIC and FORMOSAT-7/COSMIC-2 is provided by Taiwan Analysis Center for COSMIC (TACC) (https://tacc.cwb.gov.tw/v2/en/index.html). The lunar phase is obtained from Lunar Calendar (https://www.timeanddate.com/).
