# Supplementary material for: The three-dimensional plasma structures and flows of the Earth’s upper atmosphere due to the Moon’s gravitational force
Source: Sci Rep. 2022 Dec 5;12:21003. doi: 10.1038/s41598-022-25449-y (PMC9722666; doi:10.1038/s41598-022-25449-y)
Supplement: Supplementary file 1 — Supplementary Information 1. [file 41598_2022_25449_MOESM1_ESM.docx]

Supplementary Material for “The three-dimensional plasma structures and flows of the Earth’s upper atmosphere due to the Moon’s gravitational force”

Jann-Yenq Liu^1,2,3*^, Tsung-Yu Wu^1,2^, Chi‐Yen Lin^1,2^, and Loren C. Chang^1,2^

^1^Center for Astronautical Physics and Engineering, National Central University, Taiwan. ^2^Department of Space Science and Engineering, National Central University, Taiwan.

^3^Center for Space and Remote Sensing Research, National Central University, Taiwan

Email: [jyliu@jupiter.ss.ncu.edu.tw](mailto:jyliu@jupiter.ss.ncu.edu.tw)

**Note on Supplemental Material**

The following sections are included:

1. Sketches of the six FORMOSAT-3/COSMIC micro satellites orbiting at the 800 km altitude with 72-deg inclination: Figure S1.
2. Radio occultation sounding of the Earth’s ionosphere and atmosphere: Figure S2
3. Sketches of six FORMOSAT-7/COSMIC-2 small satellites at 550 km altitude with 24-deg inclination: Figure S3
4. FORMOSAT-3/COSMIC and FORMOSAT-7/COSMIC-2 observations and coverages: Figure. S4
5. The hmF2 on the equatorial plane on the 0/15, 4/18, 7/22, and 12/26 days: Figure S5
6. hmF2 and NmF2 during various solar activity and the geomagnetic conditions: Figure S6
7. Electron density observed by the F3/C and F7/C2 at various lunar local time and altitude during 2006–2020: Figure S7
8. Altitude-magnetic latitude slices of the electron density at various LLT: Figure. S8
9. A sketch of the response of electron density profiles to verticle motions: Figure. S9
10. A sketch of using the Abel transform to obtain a vertical profile of electron density: Figure. S10


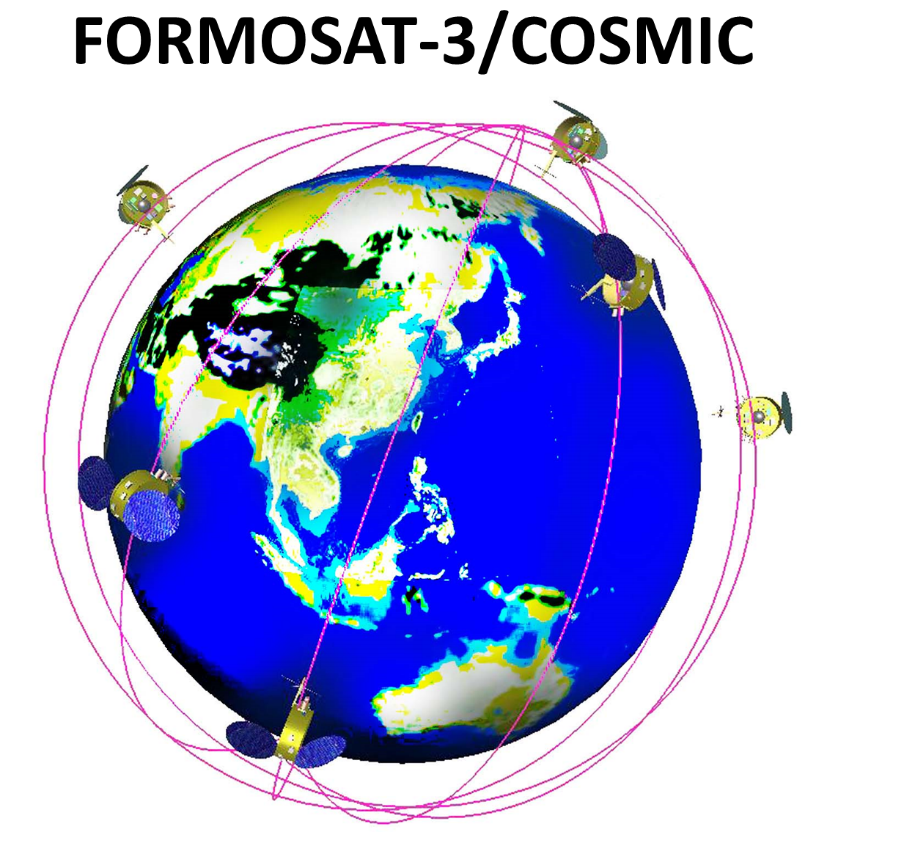


**Figure S1. Sketches of the six FORMOSAT-3/COSMIC micro satellites orbiting at the 800 km altitude with 72-deg inclination.** The F3/C satellites are sketched by National Space Organization (NSPO, <https://www.nspo.narl.org.tw/>) using Photoshop CS6 and Illustrator CS6.


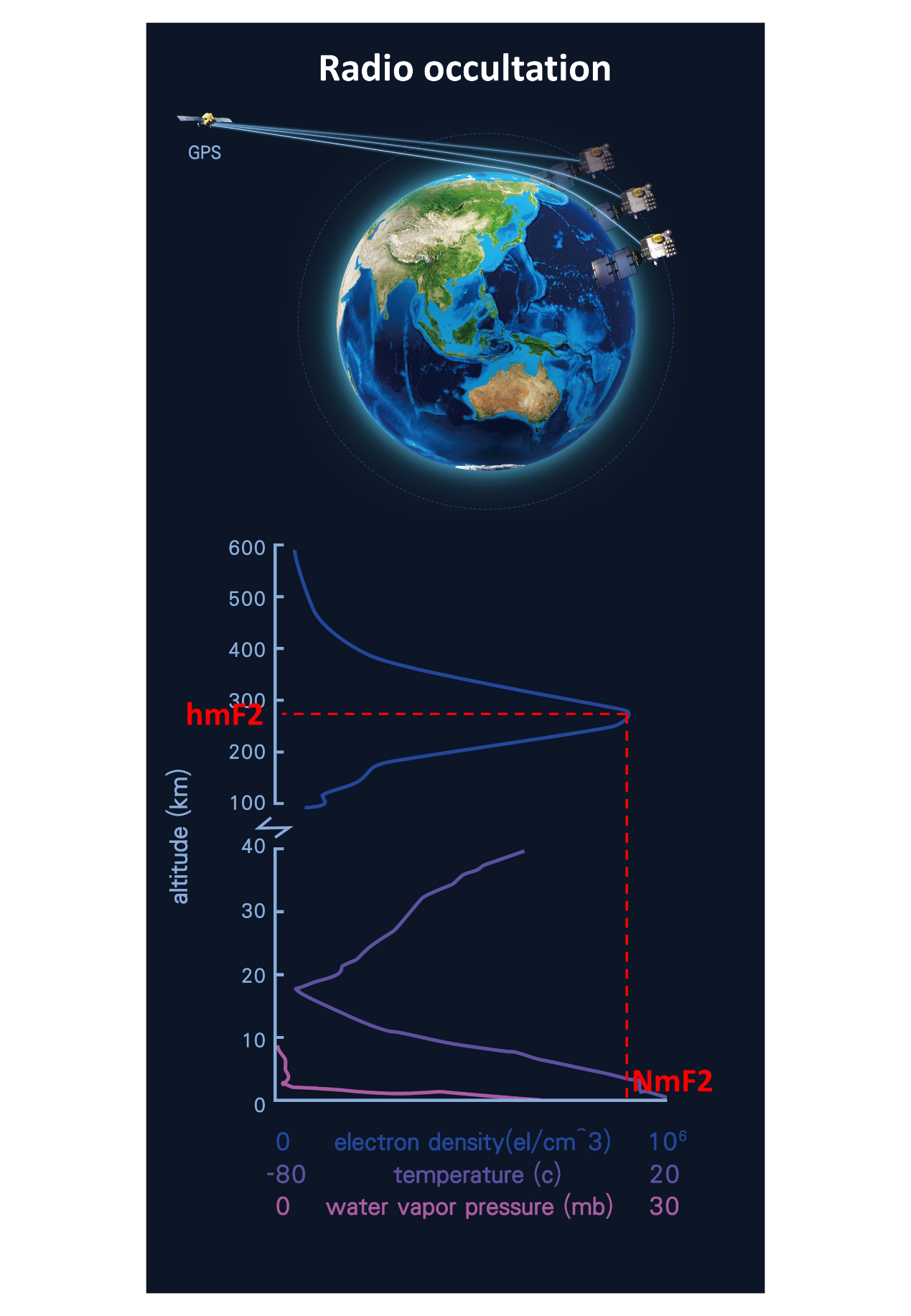


**Figure S2. Radio occultation sounding of the Earth’s ionosphere and atmosphere.** RO sounding images are sketched by National Space Organization (NSPO, <https://www.nspo.narl.org.tw/>).


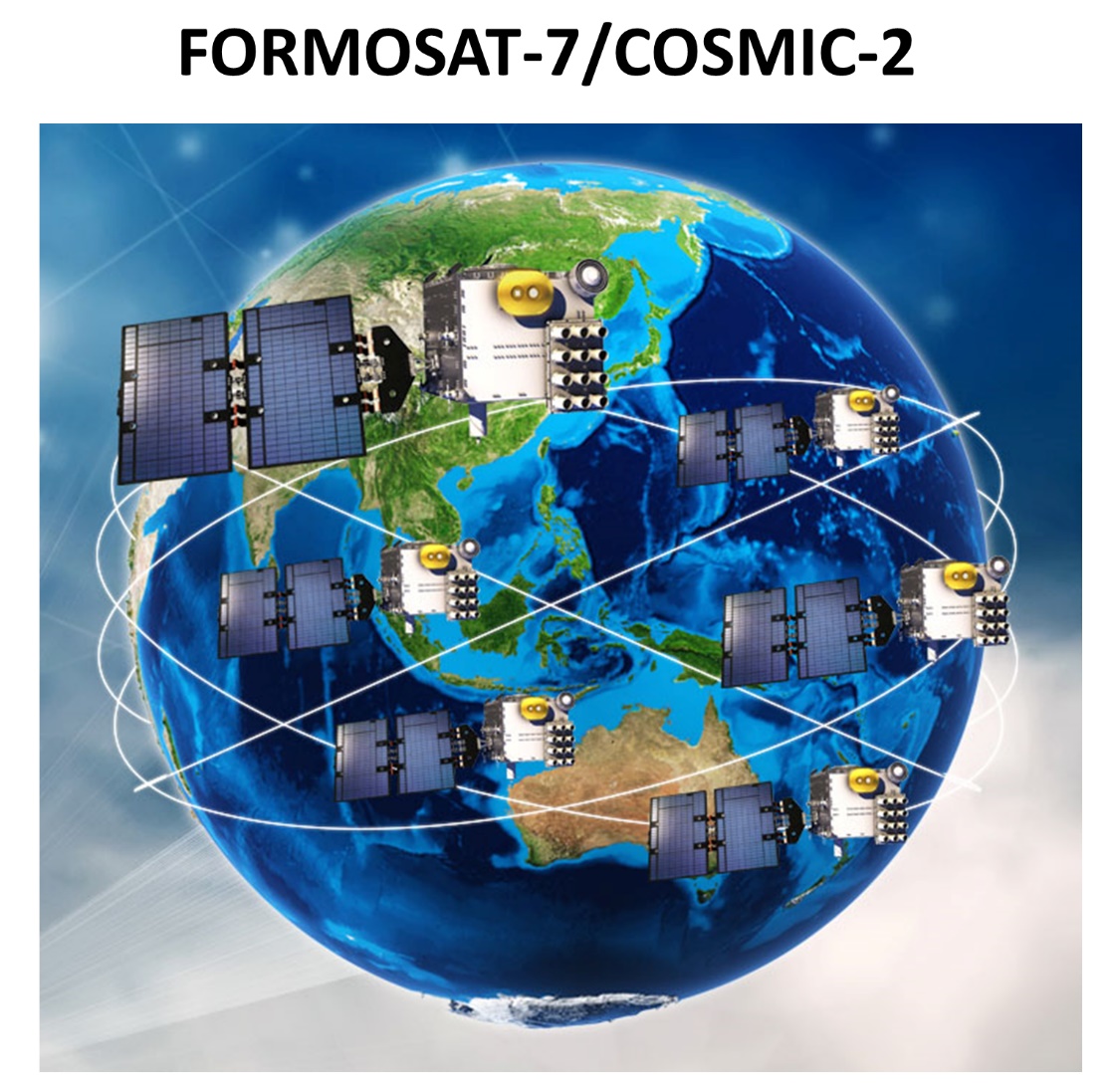


**Figure S3. Sketches of six FORMOSAT-7/COSMIC-2 small satellites at 550 km altitude with 24-deg inclination.**  The F7/C2 satellites are sketched by National Space Organization (NSPO, <https://www.nspo.narl.org.tw/>) using Photoshop CS6 and Illustrator CS6. The F7/C2 satellite image is credited by Surrey Satellite Technology (SSTL, <https://www.sstl.co.uk/>).


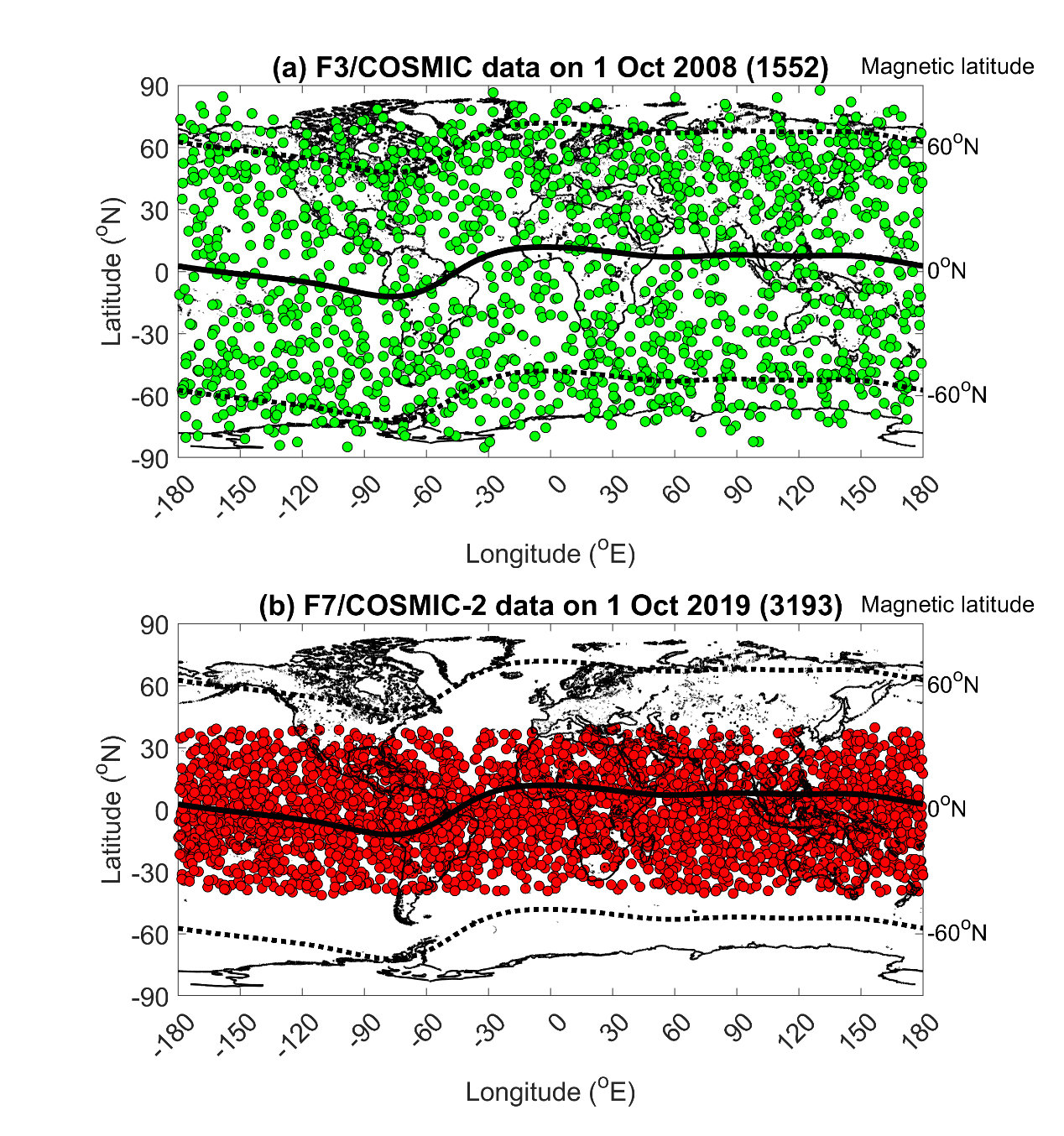


Figure S4. FORMOSAT-3/COSMIC and FORMOSAT-7/COSMIC-2 observations and coverages. (a) 1552 and (b) 3193 ionospheric RO profiles sounded daily by F3/C and F7/C2, respectively.


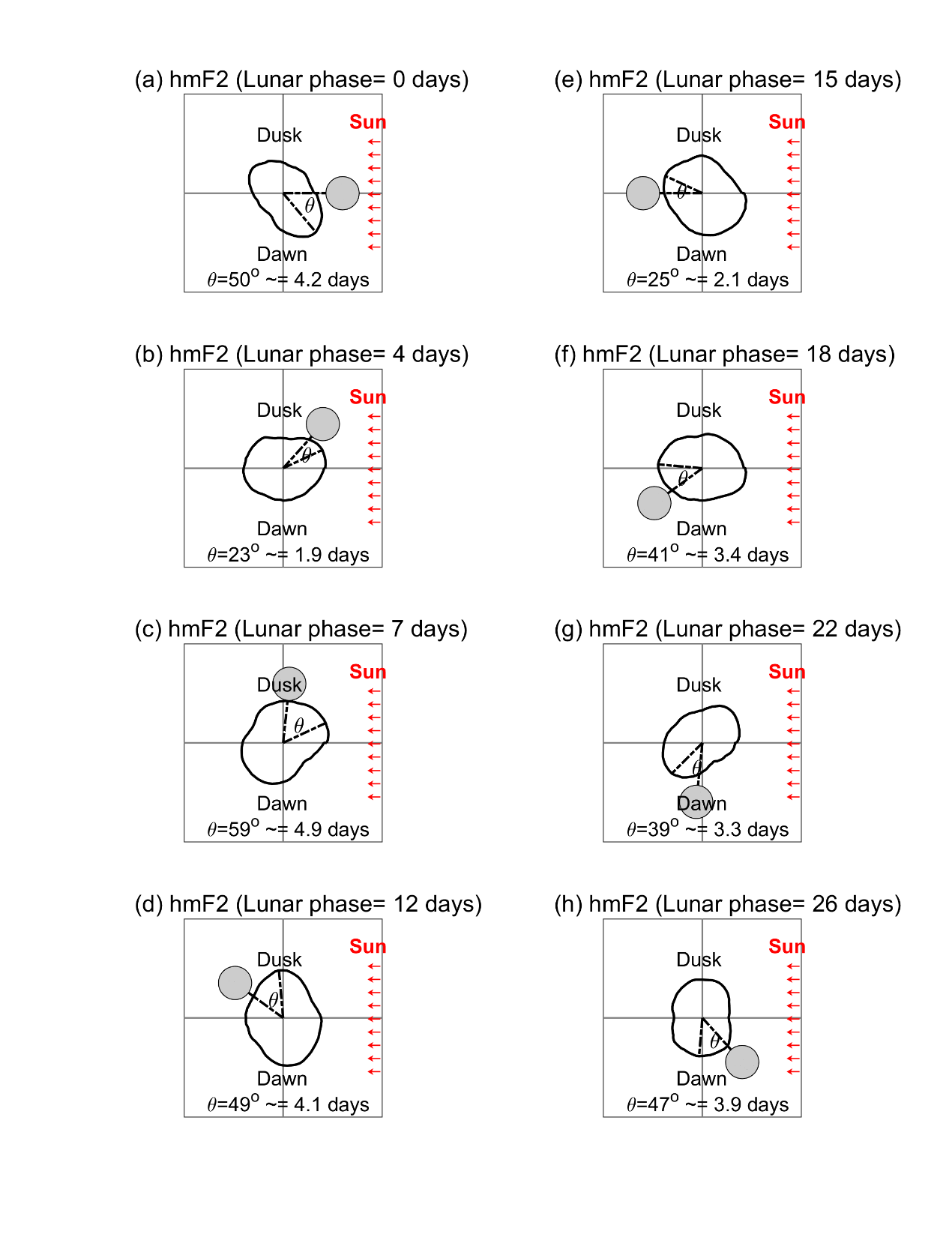


Figure S5 The hmF2 on the equatorial plane on the 0/15, 4/18, 7/22, and 12/26 days.

To study the double bulges signature detailed, the hmF2 and NmF2 during various solar activity and the geomagnetic conditions are examined. Figure S6 displays the magnetic latitude-LLT maps of hmF2, NmF2, ΔhmF2 and ΔNmF2 during entire 15-year, storm‐free (i.e., without storms; Dst > −50 nT), as well as high (F10.7 ≥ 100 sfu) and low (F10.7 < 100 sfu) solar activity periods. The hmF2 (1^st^ column of Fig. S6), NmF2 (2^nd^ column of Fig. S6), ΔhmF2 (3^rd^ column of Fig. S6), and ΔNmF2 (4^th^ column of Fig. S6) respectively present very similar patterns, except for those during the high solar activity period (3^rd^ row of Fig. S6). It can be seen that hmF2 ascends to higher altitudes (325-350 km) and NmF2 reach greater values (9$\times$10^5^ #/cm^3^), both slightly expend toward higher latitudes during the high solar activity (Fig. S6). Nevertheless, prominent double bulge features can be observed under virous magnetic storm conductions and solar activities.


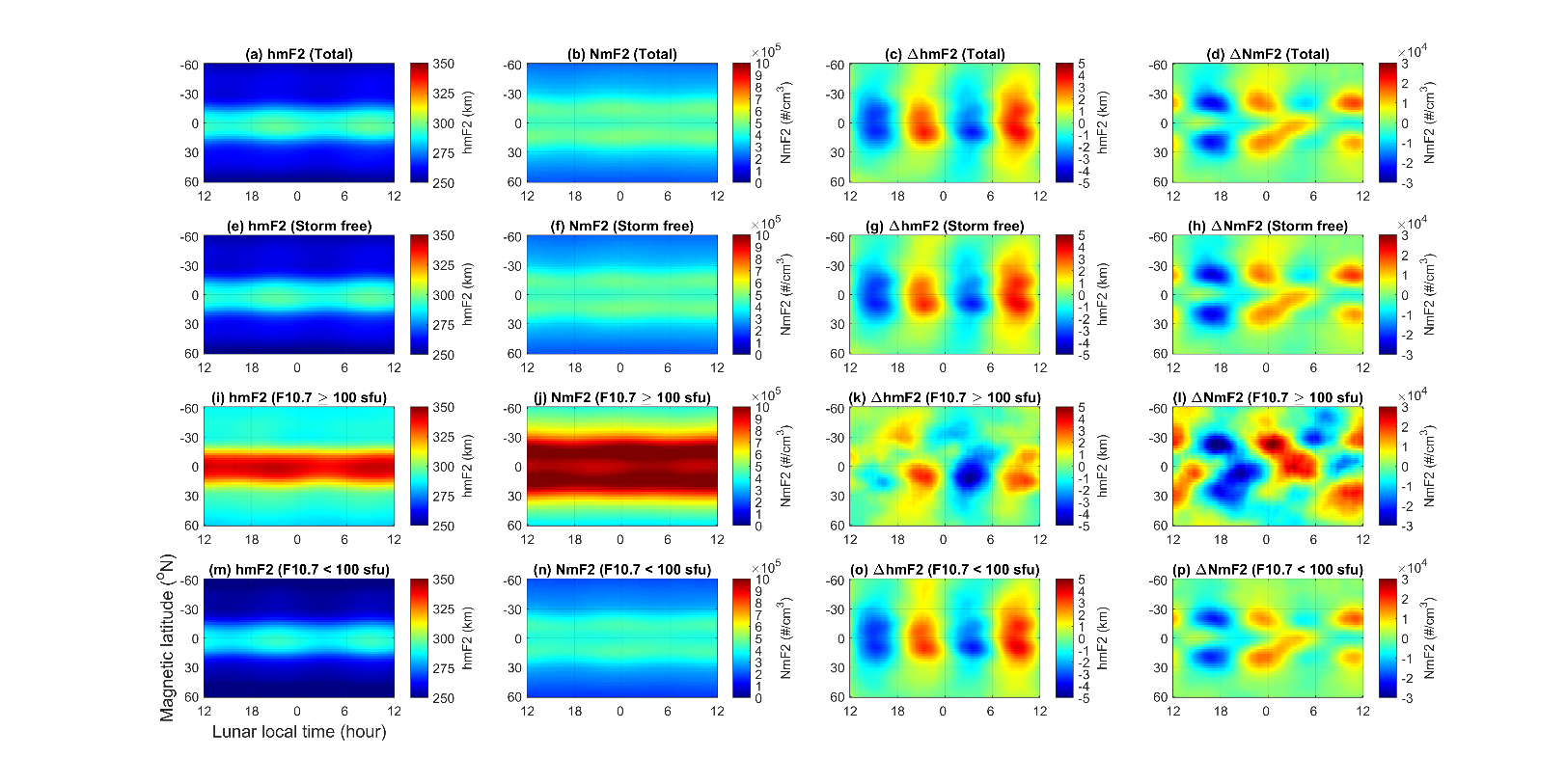


**Figure S6. hmF2 and NmF2 during various solar activity and the geomagnetic conditions.**  hmF2, ΔhmF2, NmF2, and ΔNmF2 during the (a–d) entire 15-year, (e–h) storm‐free, (i–l) high, and (m–p) low solar activities.


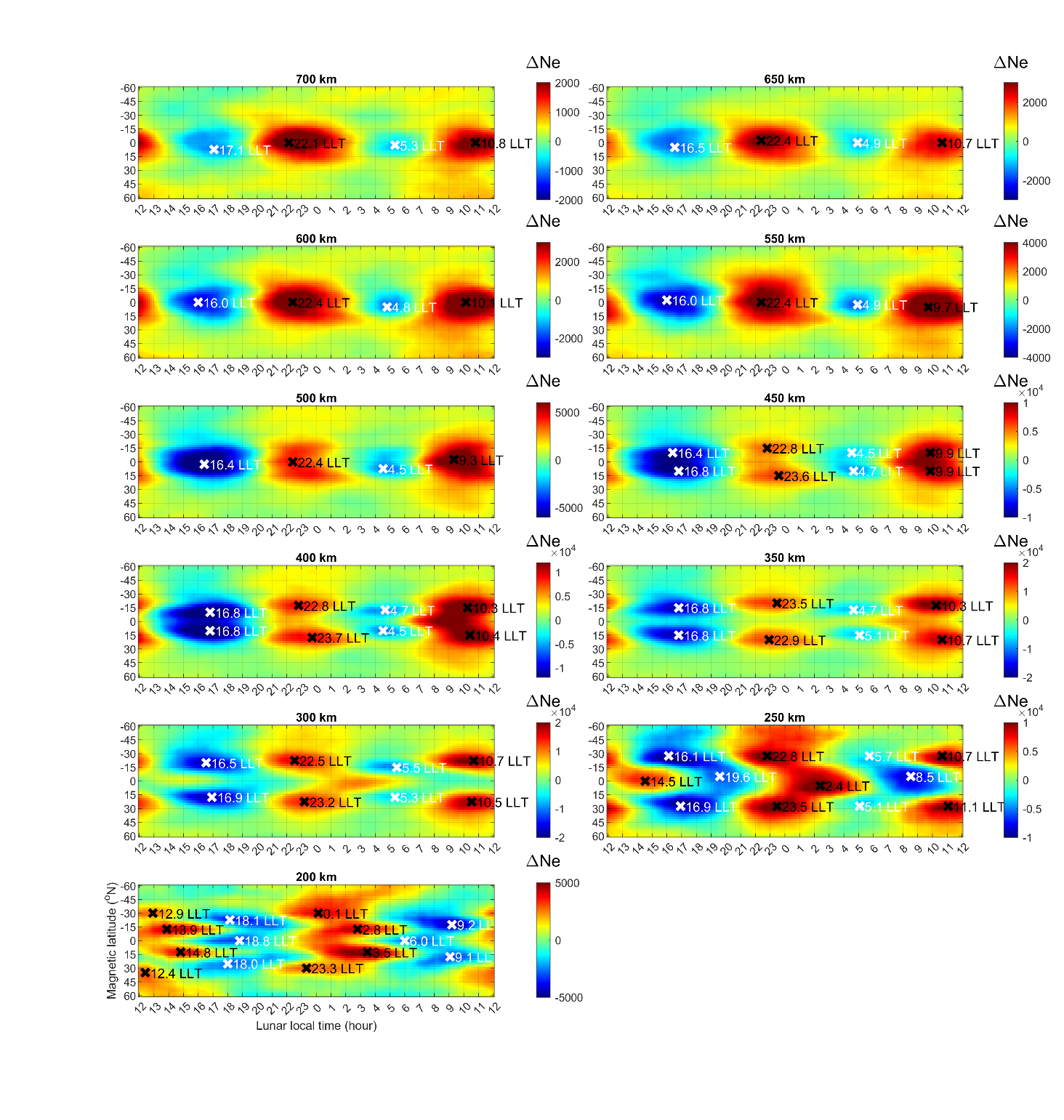


**Figure S7. Electron density observed by the F3/C and F7/C2 at various lunar local time and altitude during 2006–2020.** The black and white cross indicate the high tide and low tide, respectively.


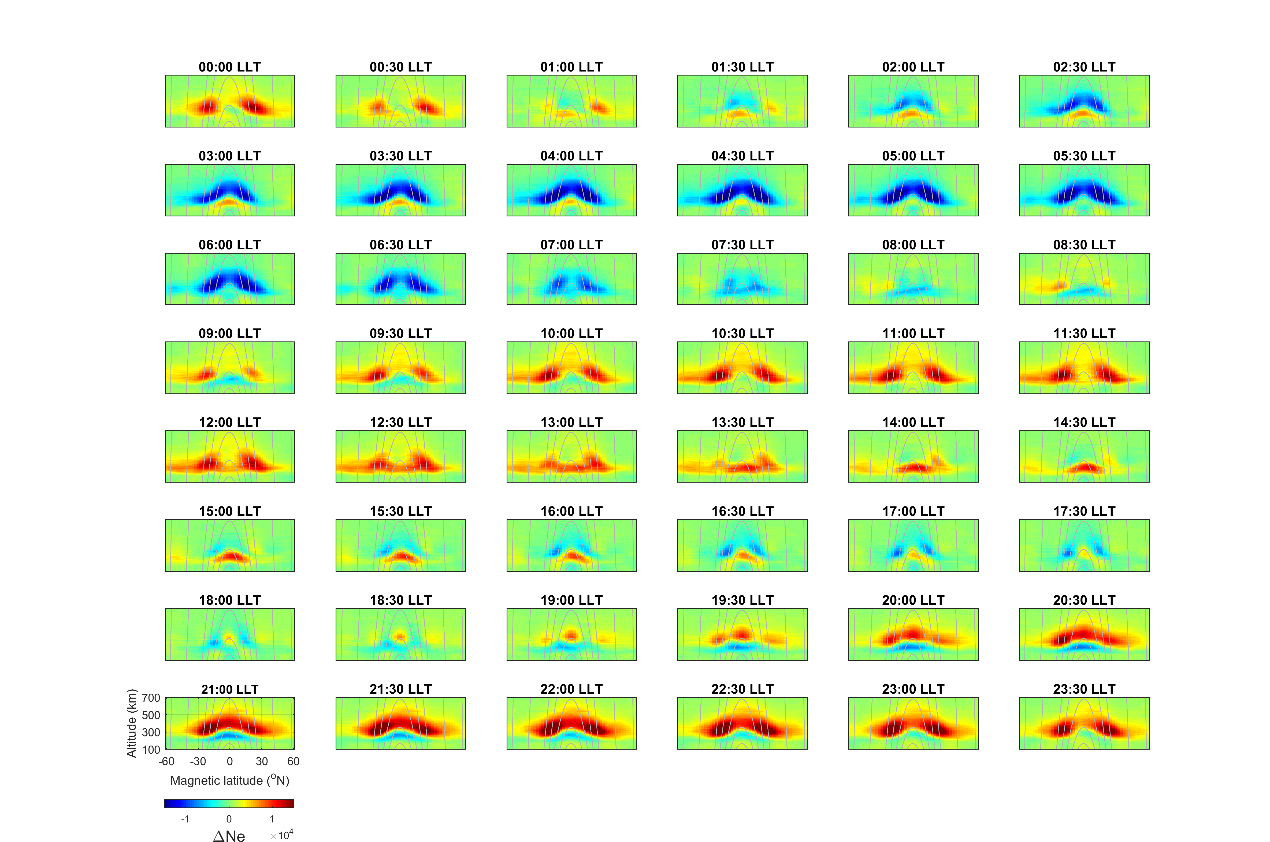
 Figure S8. Altitude-magnetic latitude slices of the electron density at various LLT. The gray curves denote the magnetic field lines.


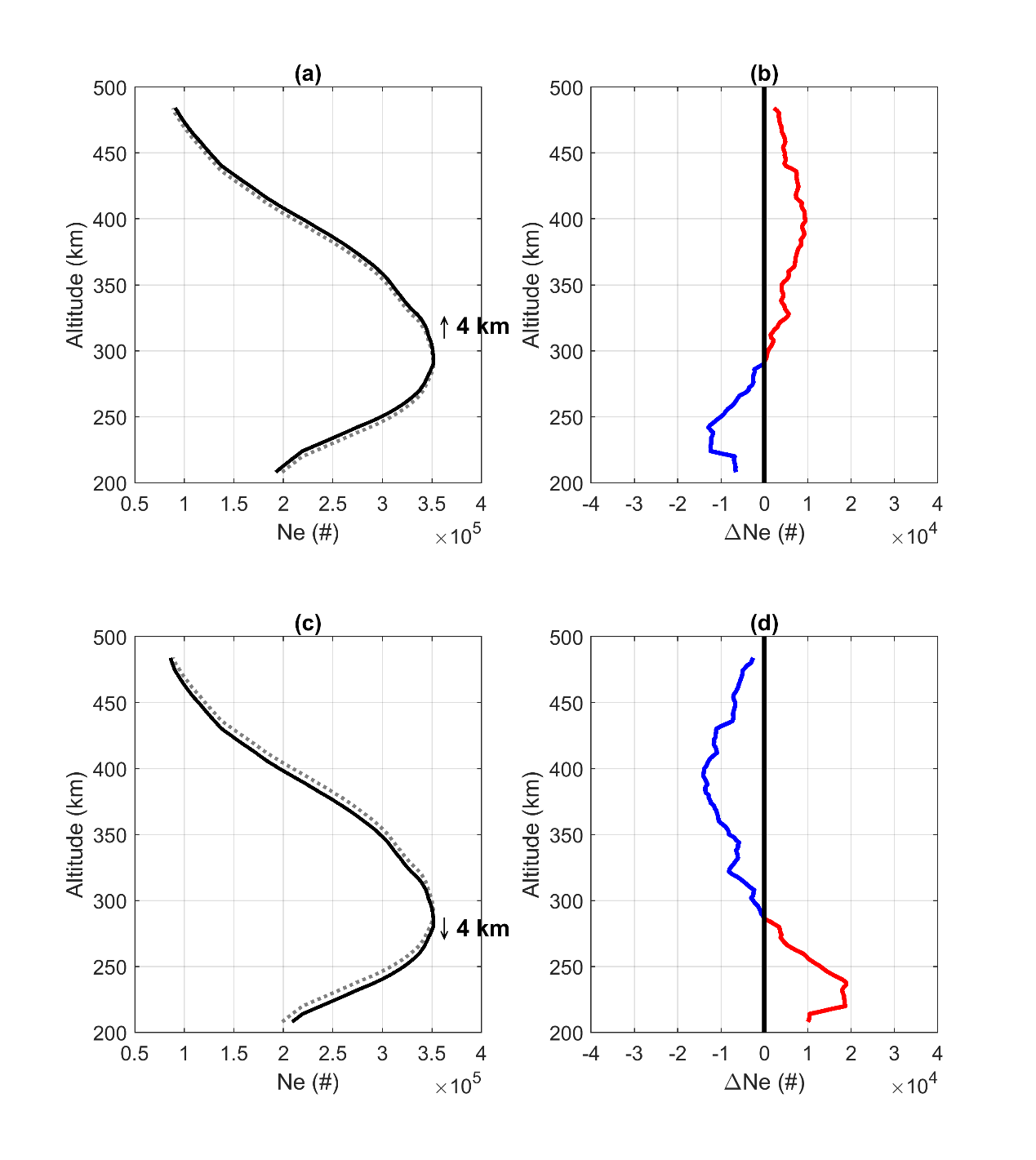
 Figure S9. A sketch of the response of electron density profiles to verticle motions. (a-b) upward and (c-d) downward motions.


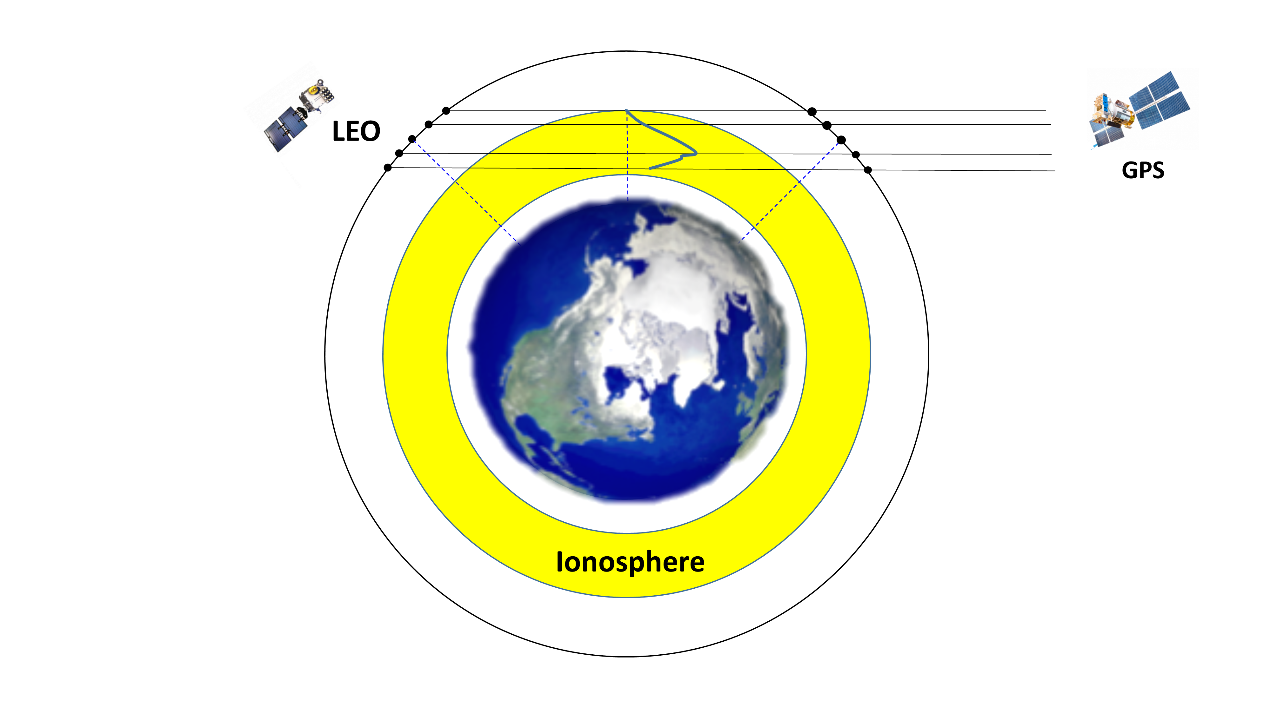


Figure S10. A sketch of using the Abel transform to obtain a vertical profile of electron density. The GPS and LEO satellite image is adopted from NSPO website (<https://www.nspo.narl.org.tw/>). The Global View of the Arctic Ocean is published by NASA/JPL/ASF (https://www.jpl.nasa.gov/images/pia02970-global-view-of-the-arctic-ocean)
