# Supplementary figures and images for: The three-dimensional plasma structures and flows of the Earth’s upper atmosphere due to the Moon’s gravitational force
Source: Sci Rep. 2022 Dec 5;12:21003. doi: 10.1038/s41598-022-25449-y (PMC9722666; doi:10.1038/s41598-022-25449-y)

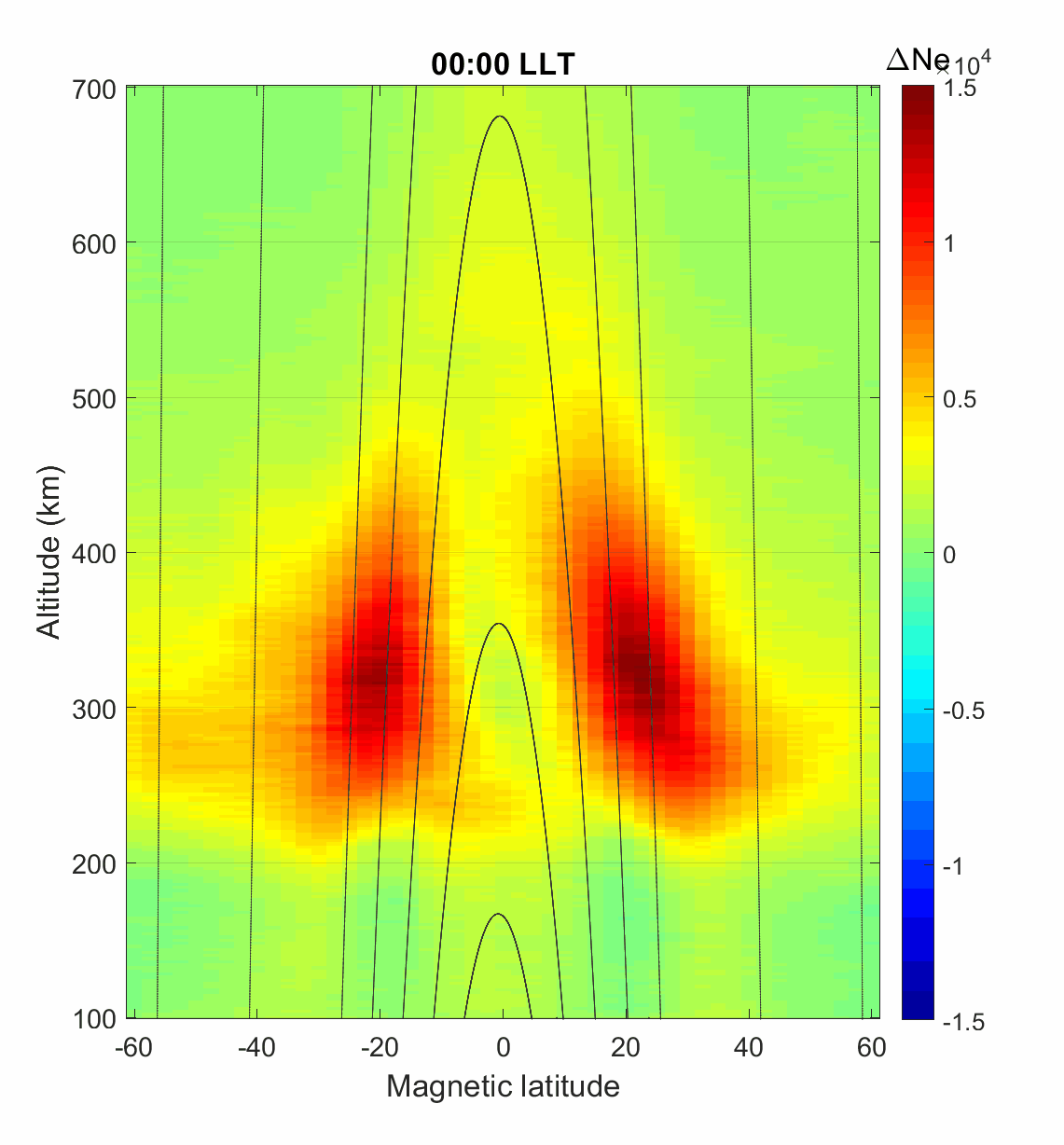

Supplement: Supplementary file 2 — Supplementary Information 2. [file 41598_2022_25449_MOESM2_ESM.gif]
